# Identification of the Rheumatoid Arthritis Shared Epitope Binding Site on Calreticulin

**DOI:** 10.1371/journal.pone.0011703

**Published:** 2010-07-22

**Authors:** Song Ling, Andrew Cheng, Paul Pumpens, Marek Michalak, Joseph Holoshitz

**Affiliations:** 1 Department of Internal Medicine, University of Michigan School of Medicine, Ann Arbor, Michigan, United States of America; 2 Latvian Biomedical Research and Study Center, Riga, Latvia; 3 Department of Biochemistry, University of Alberta, Edmonton, Alberta, Canada; University of Cambridge, United Kingdom

## Abstract

**Background:**

The rheumatoid arthritis (RA) shared epitope (SE), a major risk factor for severe disease, is a five amino acid motif in the third allelic hypervariable region of the HLA-DRβ chain. The molecular mechanisms by which the SE affects susceptibility to – and severity of - RA are unknown. We have recently demonstrated that the SE acts as a ligand that interacts with cell surface calreticulin (CRT) and activates innate immune signaling. In order to better understand the molecular basis of SE-RA association, here we have undertaken to map the SE binding site on CRT.

**Principal Findings:**

Surface plasmon resonance (SPR) experiments with domain deletion mutants suggested that the SE binding site is located in the P-domain of CRT. The role of this domain as a SE-binding region was further confirmed by a sulfosuccinimidyl-2-[6-(biotinamido)-2-(*p*-azido-benzamido) hexanoamido] ethyl-1,3-dithiopropionate (sulfo-SBED) photoactive cross-linking method. *In silico* analysis of docking interactions between a conformationally intact SE ligand and the CRT P-domain predicted the region within amino acid residues 217–224 as a potential SE binding site. Site-directed mutagenesis demonstrated involvement of residues Glu^217^ and Glu^223^ - and to a lesser extent residue Asp^220^ - in cell-free SPR-based binding and signal transduction assays.

**Significance:**

We have characterized here the molecular basis of a novel ligand-receptor interaction between the SE and CRT. The interaction represents a structurally and functionally well-defined example of cross talk between the adaptive and innate immune systems that could advance our understanding of the pathogenesis of autoimmunity.

## Introduction

The “shared epitope” (SE) is a five amino acid sequence motif in positions 70–74 of HLA-DRβ chains encoded by *HLA-DRB1* alleles that are strongly associated with susceptibility to severe rheumatoid arthritis (RA). The mechanism underlying SE-RA association is unclear. Based on the known role of MHC class II molecules in presentation of antigenic peptides to helper T cells, it has been hypothesized over the past two decades that RA-SE association is due to presentation of arthritogenic self or foreign peptides [1], [2]. However, this theory is difficult to reconcile with lack of conclusive evidence to support antigen-specific responses as the primary event in RA, the promiscuous association of the SE with other human diseases and various autoimmunity models in different species, plus the unexplained SE gene-dose effect on disease severity and penetrance (reviewed in [3]).

Based on our recent data [4], [5], [6], we have proposed an alternative hypothesis, postulating that the SE, analogous to certain domains of class I MHC-molecules [7], [8], acts as an innate immune system ligand. We have demonstrated that the SE acts as a signaling ligand in its native conformation within cell surface-expressed HLA-DR molecules, as well as a cell-free HLA-DR tetrameric molecule. The activity could also be observed when the ligand was genetically engineered into non-HLA recombinant proteins, or as a short synthetic peptide. In all these configurations, the SE activated robust production of nitric oxide (NO) and reactive oxygen species (ROS) in other cells [4], [5], [6].

In previous studies [6] we have shown that SE-activated signaling depends on cell surface calreticulin (CRT). The affinity of SE-CRT interaction was calculated to be at a low-µM range, similar to many other receptor-ligand interactions in the immune system. CRT is critical for SE-triggered signaling, as anti-CRT antibodies and small interfering RNA oligonucleotides blocked SE-activated signaling and murine embryonic fibroblasts (MEF) from *crt* ^−/−^ mice failed to transduce SE-activated signals. However, when soluble CRT was exogenously added to *crt* ^−/−^ cells, it attached to the cell surface and restored signaling responsiveness [6]. Thus, SE-activated signaling depends on binding to cell surface CRT, which plays a critical role in signal transduction.

CRT is an endoplasmic reticulum chaperone, which also functions as a co-receptor when expressed on the cell surface [9]. It has long been known to function as an innate immune system receptor. For example, CRT has been implicated as a receptor for C1q, mannose binding lectin and members of the collectin family [10], [11]). Due to its critical importance for elimination of apoptotic cells [12], CRT is believed to play a pivotal role in the junction between tolerance and autoimmunity [13]. Aberrant activation of the CRT-mediated pathway can lead to autoimmunity, as exemplified by conditions that involve defective CRT-mediated clearance of apoptotic cells [14]. CRT has been previously hypothesized to play a role in autoimmune human diseases, including RA [15]. Thus, our findings that CRT serves as the signal transduction receptor for SE, a major factor in RA disease susceptibility and severity, could provide important new insights into the role of CRT in autoimmunity. To gain better understanding of SE-CRT interaction, here we have undertaken to map the SE binding site on CRT.

## Results

### Identification of the SE-binding CRT domain

**Figure 1:**
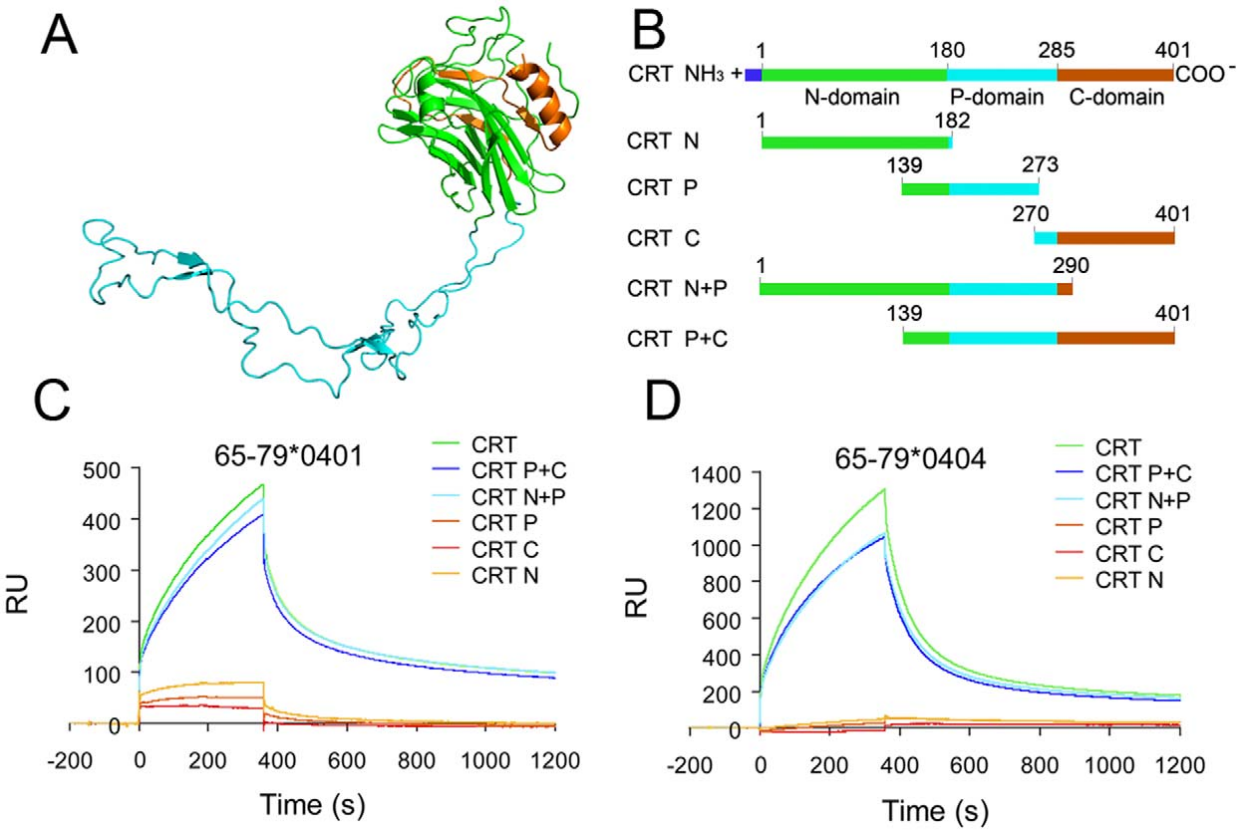
Interaction between the SE and domain-deleted CRT. **A.** A theoretical 3D structure of CRT. Rabbit CRT (Accession No. AAB20096) was computationally modeled based on the known crystal structure of Calnexin (PDB ID: 1JHN), using Modeler 9v7 software (School of Pharmacy, UCSF). CRT N-domain is shown in green, P-domain is in cyan and C-domain is shown in orange. **B**. Schematic representation of rabbit CRT domain-deletion mutants. **C,D.** SPR-based interactions between the SE ligand and CRT domain-deletion mutants. WT CRT or its domain-deletion mutants were immobilized on a CM5 biosensor chip surface and the SE peptidic ligands 65-79*0401 (**C**) or 65-79*0404 (**D**) were run in the analyte.

The primary sequence of CRT suggests that similar to calnexin it has three domains [16]. Because the tri-dimensional structure of CRT has not been fully resolved, it is a common practice in the literature to model it based on the known crystal structure of calnexin [17]. According to this model (Fig. 1A) the amino-terminal segment (N-domain) has a globular β-sheet structure. This domain is followed by a proline-rich sequence, called the P-domain that folds into a long hairpin-like structure with two pairs of short anti-parallel β-sheet sequences. The third region of CRT, called the C-domain, forms, together with the N-domain, a globular “head” to the molecule.

To determine to which of these three domains the SE binds, we first used domain deletion mutants that code truncated segments of CRT (Fig. 1B). The recombinant products of these mutants were immobilized on a CM5 biosensor chip and their binding to SE-expressing 15mer peptides was assayed as previously described [6]. As can be seen in the Figures 1C and 1D, consistent with our previous report [6], SE-expressing peptides 65-79*0401 and 65-79*0404 both interacted with the intact CRT molecule. Truncated proteins containing single domains (N, P or C-domains) failed to bind the SE. However, when truncated proteins containing the N-domain plus P-domain, or P-domain plus C-domain were tested, near-normal binding interactions could be seen. This pattern indirectly implicates the P-domain and suggests that a conformation-dependent binding site is involved.

**Figure 2:**
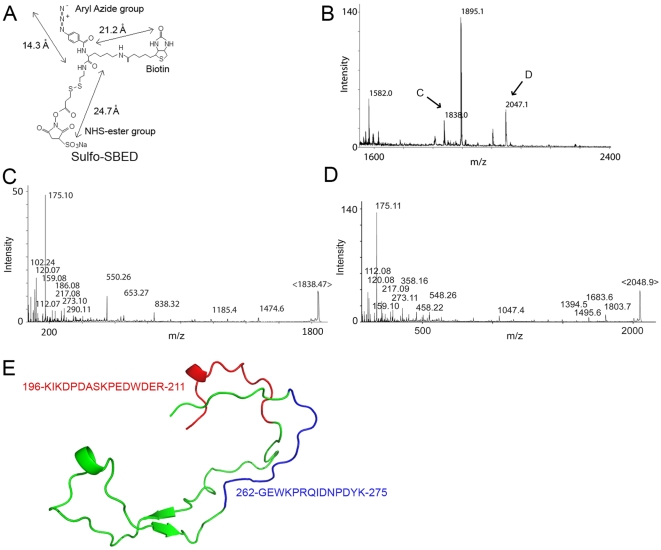
Photoactive cross-linking studies. **A**. Structural formula of Sulfo-SBED. **B**. MS analysis of trypsinized sulfo-SBED-labeled CRT fragments. **C**. MS/MS data of peak 2047.1 (marked as “C” in Fig. 2B) identifying CRT segment 196-KIKDPDASKPEDWDER-211. **D**. MS/MS data of peak 1838 (marked as “D” in Fig. 2B) identifying CRT segment 262-GEWKPRQIDNPDYK-275. **E**. Spatial proximity of peaks 2047.1 (red) and 1838 (blue), as predicted by NMR-based tri-dimensional analysis of the CRT P-domain [19].

To more directly identify the binding domain, we have used a photoactive cross-linking approach with sulfosuccinimidyl-2-[6-(biotinamido)-2-(*p*-azido-benzamido) hexanoamido] ethyl-1,3-dithiopropionate (sulfo-SBED) [18]. This cross-linker has 4 functional groups: a NHS-ester group, a UV-activatable aryl azide group, a cleavable disulfide bond, and a biotin group (Figure 2A). The NHS-ester group was chemically attached to the N-terminal amine group of SE-positive peptide 65-79*0404 and the compound was allowed to interact with CRT. Following UV cross-linking, cleavage of the disulphide bond and trypsinization, the biotinylated CRT protein digest was captured by an avidin column, and the affinity-purified biotinylated fragments were analyzed by mass spectrometry (MS). A representative experiment, one of two repetitions, is shown in Fig. 2. Four major peaks were identified (Fig. 2B). Of these, peaks 1895 and 1582 were found to correspond to contaminating non-CRT peptides (avidin and the SE peptides, respectively, data not shown). However, peaks 1838 (Fig. 2C) and 2047.1 (Fig. 2D) were found to correspond, respectively, to the 262–275 and 196–211 regions in the CRT P-domain. It is worth noting that despite a 51 amino-acid distance between the two regions, NMR data [19] indicate that they are spatially adjacent – on opposite sides of the two antiparallel arms of the P-domain hairpin structure (Fig. 2E). Given the size of the sulfo-SBED compound (Fig. 2A), when taken together, these findings strongly suggest that the SE binding site is located in the CRT P-domain, within ∼30 Å from regions 262–275 and 196–211.

### Computer simulation of SE-CRT interaction

In order to identify candidate SE binding sites on CRT P-domain, we have used the BioMedCAChe 6.1 docking software. To this end, the third allelic hypervariable region (aa 65–79) of the HLA-DR β chain, including the SE ligand region (aa 70–74) was modeled in its α helical conformation as predicted by HLA-DR1 [20] and HLA-DR4 [21] crystal structure data. The P-domain was maintained in its tri-dimensional conformation as previously predicted by NMR analysis [19]. The conformationally-rigid SE ligand and the CRT P-domain (PDB ID: 1HHN) were docked using the BioMedCAChe Augmented MM3 software. As shown in Table 1, four docking models with significant levels of docking energy were identified. Intriguingly, all four models predicted the region 217–224 as the binding site.

**Table 1:** Docking models: Interactions between SE and CRT residues.

		Residues 70–74 (DRB1*0401)				
Model	Docking score (kcal/mole)	Gln^70^	Lys^71^	Arg^72^	Ala^73^	Ala^74^
A	−232	Glu^217^		Asp^220^	Asp^220^	
		Asp^220^		Glu^223^		
				His^224^		
B	−170	Glu^223^	Glu^223^ His^224^			Asp^220^
		His^224^				
C	−166	Glu^223^				Asp^220^
		His^224^				
D	−92		Asp^220^			Asp^220^
			Glu^223^			

**Figure 3:**
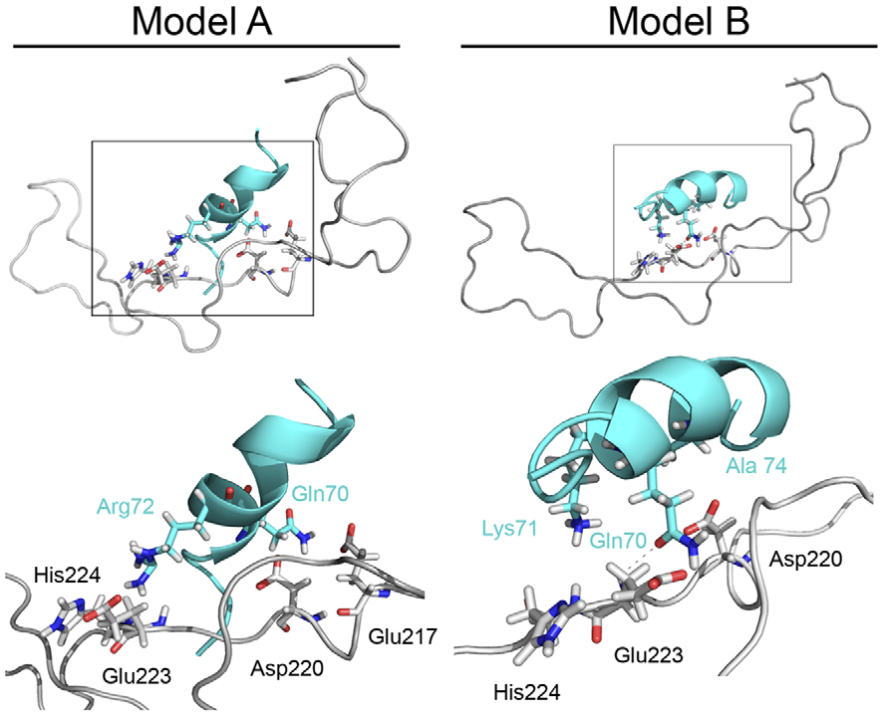
SE-CRT docking models. Graphic representation of the two most significant docking models shown in Table 1: Model A (left) and Model B (right). Closer views of the interactions between CRT (black font) and SE (cyan font) residues are shown in the lower panels.

The two most significant docking models are depicted graphically in Figure 3. As can be seen, in model A (the most significant one, with docking score of −232 kcal/mole), CRT residues Glu^217^ and Asp^220^ interact with SE residue Gln^70^, while CRT residues Glu^223^ and His^224^ interact with SE residue Arg^72^. In model B (docking score of −170 kcal/mole), CRT residue Glu^223^ is in close proximity to SE residues Lys^71^ and Gln^70^, while CRT Asp^220^ is in close proximity to SE residue Gln^70^ and Ala^74^.

### Site-directed mutational analysis

Based on the docking models mentioned above, we next performed site-directed mutagenesis in residues predicted to interact with the SE. The resultant point-mutated recombinant proteins were analyzed by circular dichroism (CD) spectroscopy and showed only minor, insignificant alterations in the secondary structure (Supplemental Figure S1).

To determine the effect of individual substitutions on CRT-SE interaction, we first used a SPR assay. Wild-type (WT) or mutant CRT proteins were immobilized on a biosensor chip at about 500 RU using a standard primary amine coupling method. SE peptidic ligands 65-79*0401 or 65-79*0404 were run as analyte. The results are summarized in Table 2. As can be seen, the CRT E217A and E223A mutants, but not mutants D220A, H224A, Y254A or N279A, showed significantly lower binding of both SE ligands. Notwithstanding the seemingly modest variations in SE-binding potency among the different CRT species, these differences were reproducible, statistically significant and, more importantly, correlated well with signaling potencies, as discussed below.

**Table 2:** SPR interactions between SE ligands and WT or mutant CRT.

	Ligand (SE)			
	65-79*0401		65-79*0404	
Receptor (CRT)	RU	% decrease	RU	% decrease
WT	71.2±5.0 (13)		127.5±10.2 (8)	
**E217A**	**50.9±1.5** ‡ (2)	28.6±2.0	**105.7±4.9** † (2)	17.1**±**3.8
D220A	70.1±6.4 (6)	1.6±9.0	119.8±13.0 (4)	6.0±10.2
**E223A**	**56.7±2.9** † (3)	20.3±4.0	**84.6±14.1** † (2)	33.6±7.9
H224A	68.2±9.8 (3)	4.1±13.7	128.4	–
Y254A	66.5	–	112.7±24.0 (2)	–
N279A	106.1	–	144.9±20.3 (2)	–

Values are shown as mean ± SEM. Significantly lower interactions are shown in bold.

^†^ , *p*<0.05;

^‡^ , *p*<0.005. The figures in parentheses represent the number of replicates.

**Figure 4:**
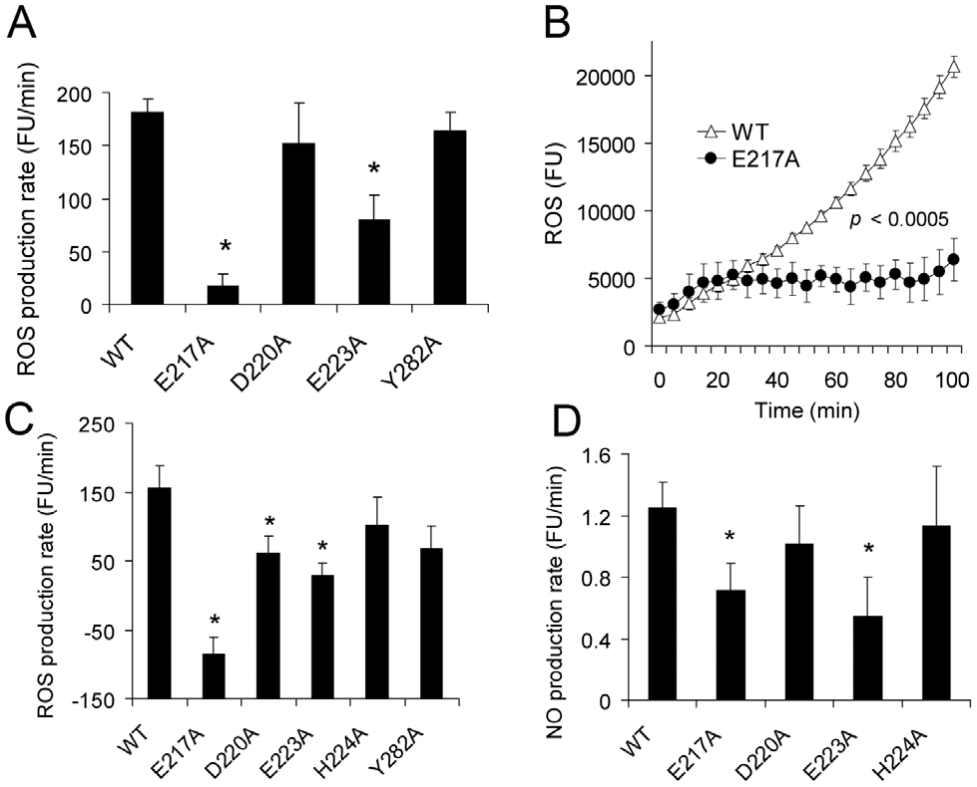
Identification of CRT residues that are critical for SE-activated signaling. **A**. K42 *crt^−/−^* MEF cells were pre-incubated overnight with 1 µg/ml of WT CRT or its mutants. Then, the SE-expressing HLA-DR tetramer T-DRB1*0401 (24 µg/ml) was added and ROS production rates were measured as fluorescent units per min (FU/minute). **B**. Representative time-course curves of ROS production in response to T-DRB1*0401 stimulation in K42 cells pre-incubated with either WT CRT (△) or CRT-E217A mutant (•). **C**. K42 *crt^−/−^* MEF cells were pre-incubated overnight with 1 µg/ml of WT CRT or its point mutants. Then, the SE-expressing ligand HBc*0401 (20 µg/ml) was added and ROS production was measured as in **A**. **D**. K42 *crt^−/−^* MEF cells were pre-incubated overnight with 1 µg/ml of WT CRT or its point mutants. Then, HBc*0401 was added and NO production rates were measured. Data are shown as incremental mean ± SEM values, above the levels obtained in the absence of the receptor and/or the ligand, as in previous studies [4]. *, *p*<0.05 compared to WT CRT.

We [6] and others [22], [23] have previously demonstrated that soluble CRT can attach to the cell surface and restore CRT receptor-mediated signaling in CRT-negative cells. Accordingly, to determine the effect of CRT point mutations on signal transduction, soluble WT or mutant CRT proteins were added to *crt^−/−^* cell line K42 [24]. There was no difference in cell surface binding capacity between WT CRT and its mutants (data not shown). As can be seen in Fig. 4A, CRT mutant E217A failed to transduce SE-activated ROS signaling and mutant E223A transduced a significantly reduced signals compared to the WT protein. No significant signaling inhibition was caused by either the D220A or Y282A mutations. Representative time-course ROS production curves with WT CRT and mutant E217A are shown in Fig. 4B. As can be seen, the E217A mutation produced complete inhibition of SE-activated ROS production. Consistent with our previous data showing close correspondence between NO and ROS signaling [4], [5], [6], Figures 4C and 4D demonstrate that the inhibitory effect of mutated residues 217 and 223 affected both NO and ROS signaling. Importantly, Figure 4 demonstrates that the inhibitory effect on SE-activated signaling by mutated residues 217 and 223 could be seen when the SE was expressed in its natural tri-dimensional conformation in the form of a tetramer (Figures 4A and 4B) or when expressed in its physiologic α helical conformation in Hepatitis B core (HBc) particles (Figures 4C and 4D). Consistent with our prior studies [6], no measurable differences in cell survival were observed in the presence or absence of WT CRT or its mutants (not shown). Although the data clearly implicate residues Glu^217^ and Glu^223^, it should be pointed out that Fig 4C suggests that, albeit weakly, residue Asp^220^ may be involved as well. Taken together, our findings indicate that SE-activated signaling maps to the 217–223 region of the CRT P-domain and depends primarily on Glu^217^ and Glu^223^ and, to a lesser extent, on Asp^220^.

## Discussion

We have previously demonstrated that the SE interacts with cell surface CRT and activates innate immune signaling [4], [5], [6]. To better characterize this interaction, here we determined the SE binding site on CRT. By using a combination of SPR-based binding studies, photoactive cross-linking methods, an *in silico* docking simulation and mutational analysis, we have mapped the SE binding site to the 217–223 region of CRT P-domain and identified residues Glu^217^ and Glu^223^ as key players.

The mature CRT protein contains three structurally and functionally distinct domains. The 180 N-terminal amino acid residues and the 115 C-terminal residues form the N-domain and a C-domain, respectively. Based on the known crystal structure of a homologous protein calnexin, these domains are predicted to fold into a composite globular domain. The intervening sequence (residues 181-284), is proline-rich and is therefore called the P-domain. This domain has been shown by NMR studies to form an arm-like hairpin structure, stabilized by two antiparallel β-sheets [19]. Consistent with our findings with domain-deleted mutants, prior studies have demonstrated that CRT tertiary conformation is critically important for its biologic activity [25].

The use of photoactive cross-linkers, followed by identification of cross-linked regions by MS analysis has proven useful in complex interactions, especially where tertiary conformational factors play a role [18], [26], [27]. Using that method, we identified two P-domain sequences located, respectively, in positions 196–211 and 262–275 of the CRT P-domain. It is worth mentioning that despite a 51 amino acid residues gap in their primary sequence, the 196–211 and 262–275 regions are spatially adjacent, on two anti-parallel segment of the P-domain hairpin. Moreover, these two regions are both within the reach of the cross-linking sulfo-SBED compound, 27.9 Å and 23.1 Å, respectively, from the SE actual binding site [19].

Our data with HLA-DR tetramers and HBc particles attest to the significance of the findings, since these physiologically-folded SE-expressing ligands closely mimic the natural conformation of the epitope. It should be mentioned that tetramers consist of four identical units of the HLA-DR molecule, each folded in its native tertiary conformation. Likewise, SE-expressing HBc particles have been engineered to allow the SE to be expressed outside of the HLA-DR context in its native α helical conformation [4]. Therefore, the significance of the point-mutant studies shown here is two-fold: First, they demonstrate high consistency between all the different mapping-approaches and, second, by using conformationally-intact reagents, these studies attest to the physiologic relevance of the data. Both SPR binding assay and signaling assay confirm that CRT Glu^217^ and Glu^223^ play critical role in binding the SE. However, we cannot rule out the possibility that other residues on other CRT domains might be involved in the binding as well. Because crystal structure data on the whole CRT molecule is unavailable, our computer docking model could only focus on the CRT P-domain.

The binding site identified here has several unique characteristics. Most of the previously reported interactions between CRT and various other proteins were found to involve its C- or N-domains, whereas little is known about the role the P-domain. Three notable exceptions in which a P-domain binding site has been implicated are ERp57 [28], [29], C1q [30], [31] and the 4-aminobutyrate type A receptor associated protein (GABARAP) [17], [32].

Using a site-directed mutagenesis approach, Martin *et al* [29] have mapped the ERp57 binding site to tip of the P-domain (residues Glu^239^, Asp^241^, Glu^243^ and Trp^244^). That binding site is different from the one reported here. Additionally, while CRT-ERp57 interaction takes place inside cells, the interaction reported here occurs on the cell surface. Cell surface CRT has been long known as an innate immune system receptor that binds and transduces C1q signals [30], [31]. The binding site of the collagen-like segment of C1q was assigned to an “S-domain”, a CRT fragment (residues 160–283), which spans both the N- and P-domains [33]. The precise site in this fragment that binds C1q has not been mapped to date and therefore its relevance to the SE binding site (residues 217–223) cannot be determined. The third known P-domain-binding ligand is GABARAP, a nervous system adaptor protein that plays a role in intracellular vesicle trafficking. Thielmann *et al* [17] have demonstrated that CRT residue Trp^183^ plays a critical role in GABARAP-CRT interaction. No site specific mutagenesis of the CRT protein was attempted in those studies. Given the location of Trp^183^ near the junction between the N-domain and P-domain, the study could not rule out participation of the N-domain in stabilizing the interaction between GABARAP and CRT [17].

It is worth mentioning that, different from GABARAP that interacts with CRT intracellularly, the SE represents an extracellular ligand that activates signaling through its interaction with cell surface CRT. Thus, the distance between Trp^183^ and the SE-binding site, the dissimilarity in the biologic effect of the two pathways and their distinct compartmentalization, all suggest that the SE and GABARAP binding sites are neither structurally nor biologically related. Thus, the SE binding site reported here is unique, both topologically and functionally.

**Figure 5:**
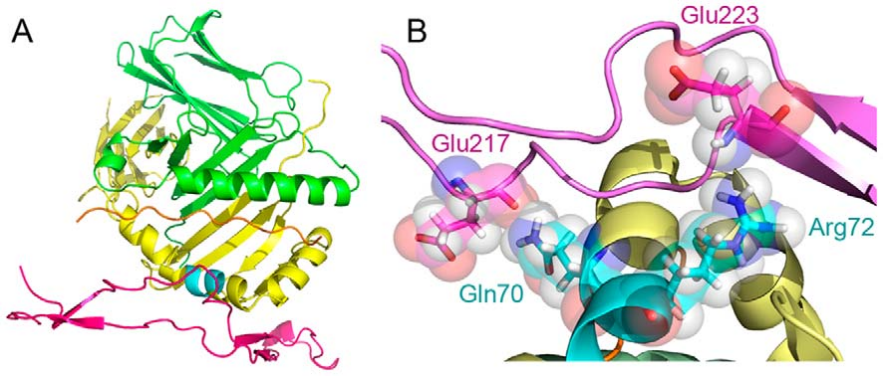
Proposed CRT-SE interaction model. **A**. A SE-positive HLA-DR4 molecule (PDB ID: 2SEB) interacting with the SE-binding site in the CRT P-domain. The HLA-DRα chain is shown in green, HLA-DRβ chain is in yellow, the SE is in cyan, CRT P-domain is in purple, and the groove peptide is shown in orange. **B**. A closer view of the interactions between CRT Glu^217^ and SE Gln^70^ and between CRT Glu^223^ and SE Arg^72^.

Based on the critical role of CRT residues Glu^217^ and Glu^223^ shown here, on the one hand and the previously determined functional role of individual SE residues on the other, Figure 5 depicts a proposed model of CRT-SE interaction. According to this model - and consistent with docking Model A discussed above -, CRT Glu^217^ interacts with SE Gln^70^, while CRT Glu^223^ interacts with SE Arg^72^. This model is supported by several considerations: 1. Model A has been assigned the most significant docking score (−232 kcal/mole); 2. Different from the other docking models considered here, it implicates Glu^217^, a residue that shows the most significant impact on both binding and signaling; 3. It involves Gln^70^ on the SE side, a residue that has been previously shown to play a critical role in RA pathogenesis [34], [35]; 4. It involves the SE Arg^72^, a residue that is common to all the SE variants (QKRAA, QRRAA and RRRAA). 5. This model allows for engagement of both the CRT P-domain and the groove peptide without spatial interference (Fig. 5). In order to examine this model, we are presently gearing towards experiments with HLA-DR tetramers carrying site-specific mutations in the SE (residues 70–74). It should be mentioned, however that while point mutation analyses on both sides will help clarifying the spatial confines of SE-CRT interaction, more definitive data might be obtained from co-crystallization studies. Unfortunately, such studies may be technically challenging, given that the crystal structure of CRT has not been resolved to this date.

In summary, we have characterized here a biologically consequential interaction site between the SE, a physiologically-folded novel extracellular ligand, and the innate immunity receptor CRT. The interaction represents a structurally and functionally well-defined example of cross talk between the adaptive and innate immune systems. In order to better understand the role of this interaction in disease pathogenesis, we are presently determining the functional consequences of SE-CRT activated pathway in the immune system. These studies could shed new light on the function of the SE and help to advance our understanding of its role in RA.

## Materials and Methods

### Cells and reagents

The mouse embryonic fibroblast (MEF) line K42, isolated from *crt* ^−/−^ mice, has been previously described [24]. pBAD-rCRT-His6, pGEX-rCRT, and its domain deletions pGEX-rCRT-N(1-182), pGEX-rCRT-P(139-273), pGEX-rCRT-C(270-401), pGEX-rCRT-N+P(1-290), and pGEX-rCRT-P+C(139-401) plasmids were prepared as described [36], [37]. Ni-NTA Superflow resin and other molecular biology reagents were purchased from QIAGEN (Valencia, CA). Diaminofluorescein Diacetate (DAF-2 DA) was purchased from EMD-Calbiochem (Gibbstown, NJ), and 5-(and-6)-chloromethyl-2′, 7′ dichlorodihydrofluorescein (CM-H_2_DCFDA) was purchased from Invitrogen (Carlsbad, CA). SE ligands expressed as either 15mer synthetic peptides (65-79*0401 or 65-79*0404), engineered into hepatitis B core particles (HBc*0401) or in the form of HLA-DR tetramers (T-DRB1*0401) were prepared as previously described [4], [5], [6]. All other chemicals were from Sigma (St. Louis, MO). Mouse *crt* cDNA was generated from brain tissue of DBA/1 mice using standard techniques. DNA sequence was confirmed with the GenBank Database (Accession no. AK075605).

### Surface plasmon resonance (SPR)

SPR experiments were performed with a BIAcore2000 instrument (GE Healthcare, Piscataway, NJ) as we described [6]. All assays were performed at 25°C in a binding buffer containing 10 mM HEPES, pH 7.4, 50 mM KCl, 0.5 mM CaCl_2_, 100 µM ZnCl_2_, and 0.005% surfactant P-20. The analyte was injected at a flow rate of 10 µl/min.

### Photoactive cross-linking studies

The sulfosuccinimidyl-2-[6-(biotinamido)-2-(*p*-azido-benzamido) hexanoamido] ethyl-1,3-dithiopropionate (Sulfo-SBED) method was used according to the manufacture's (Thermo Scientific, Waltham, MA) instructions. Briefly, the 15mer peptide 65-79*0404 was labeled with Sulfo-SBED in an equal molar ratio, using the ProFound™ Sulfo-SBED Biotin Label Transfer Kit (Thermo Scientific). Extra Sulfo-SBED was removed by gel filtration. Peptide-protein interaction was performed by incubating Sulfo-SBED-labeled 65-79*0404 with purified rabbit CRT in the dark at room temperature for 1 hr. Cross linking was achieved by UV irradiation using a Philips UV lamp (360 nm, 40 W, 3 D system PCA) for 15 minutes on ice at a distance of 5 cm. After cleaving the disulfide bond by DTT, the Sulfo-SBED-cross-linked CRT was trypsinized overnight at 37°C. Biotin-labeled peptide fragments were then isolated by a monomeric avidin column.

Mass spectrometric identification of biotin-labeled peptide fragments was performed in the University of Michigan Proteome Mapping Core. Briefly, 5 µl of α-cyano-4-hydroxycinnaminic acid (5 mg/ml in 50% acetonitrile, 0.1% TFA, 2 mM ammonium citrate) matrix was added to the 30 µl of concentrated biotinylated peptides. The samples were brought to dryness and 5 µl of 50% acetonitrile/0.1% TFA were added back into the well. 0.5 µl of this solution was hand-spotted on a 192-well target plate and allowed to dry. MALDI TOF/TOF mass spectra were obtained using an Applied Biosystems 4800 Proteomics Analyzer. Peptide masses were acquired for the range of 800–3500 Da. Mass spectra were summed from 2,000 laser shots from an Nd-YAG laser operating at 355 nm and 200 Hz. Three trypsin autolysis peaks were used for internal calibration. MS/MS spectra were acquired in MS/MS 2 kV Positive mode. Spectra were acquired for 6,000 laser shots, or until 5-peptide fragment ions reached a signal-to-noise ratio of 100. Fragmentation of the peptides was induced by the use of atmosphere as a collision gas with a pressure of ∼6×10^−7^ torr and collision energy of 2 kV. Database searching was performed using Applied Biosystems GPS Explorer v. 3.6, with Mascot v. 2.1.

### Docking models

The BioMedCAChe 6.1 software (Fujitsu, Sunnyvale, CA) was used for *in silico* modeling of the interaction between the SE and the CRT P-domain. The β-chain third allelic hypervariable regions (residues 65–79) of HLA-DR1 (PDB ID: 1T5X) and HLA-DR4 (PDB ID: 2SEB) were used as ligands and the rat CRT P-domain (PDB ID: 1HHN) was used as the receptor. Both binding partners were modeled in their rigid conformation, using augmented MM3 parameters [38]. Docking scores were calculated using the manufacturer's software. In all cases, the minimum potential energy was calculated for the most stable geometry.

### Site-directed mutagenesis

CRT site-directed mutants were generated following the QuikChange protocol (Stratagene La Jolla, CA). The primers used in this study are listed in supplemental Table S1. Sequences were verified by the University of Michigan DNA Sequencing Core. Rabbit and mouse wild-type CRT and site-directed mutants were constructed in pBAD vector. Wild-type and mutant CRT plasmids were transformed into GC10 cells for protein expression. The 6×His-tagged protein expression was induced by 0.002% L-arabinose for 4 hr and the protein was purified using a Ni-NTA resin, following the manufacturer's (QIAGEN, Valencia, CA) protocol. Rabbit CRT domain-deletion mutants were constructed in a pGEX vector. GST-fusion CRT domain-deletion mutants were expressed and purified as previously reported [36], [39]. The GST segment was removed by Factor Xa.

### Circular dichroism spectroscopy

Proteins were in a 5 mM HEPES (pH 7.4), 100 mM KF, 2 mM CaCl_2_ buffer. CD spectra were determined in 1 mm bandpass quarta cuvettes by far-UV circular dichroism on an Aviv 215 spectropolarimeter (Aviv Associates, Lakewood, NJ). Corresponding baseline levels were obtained with buffer only and subtracted from the sample spectrum.

### Signal transduction assays

To measure NO production, *crt^−/−^* MEF, 30,000 cells per well, were seeded in a 96-well plate. Cultures were loaded with 1 µg/ml of recombinant WT or mutant CRT overnight. Cells were then labeled by 20 µM DAF-2 DA and stimulated with SE ligands. NO production rates were determined as we previously described [4]. ROS production was quantified similarly, with the exception that cells were labeled with 10 µM CM-H_2_DCFDA as we previously described [5].

## Supporting Information

Figure S1 CD spectra of WT CRT and its mutants. Proteins, in a 5 mM HEPES (pH 7.4), 100 mM KF, 2 mM CaCl2 buffer were placed in 1 mm bandpass quarta cuvettes and analyzed by far-UV circular dichroism on an Aviv 215 spectropolarimeter (Aviv Associates, Lakewood, NJ). Corresponding baselines were obtained with buffer which were subtracted from the sample spectrum. No statistically significant conformational differences between WT CRT and its mutants were found.
(0.26 MB DOC)

Table S1 Primers for rabbit and mouse CRT site-directed mutants.
(0.06 MB DOC)
