# Supplementary figures and images for: Identification of the Rheumatoid Arthritis Shared Epitope Binding Site on Calreticulin
Source: PLoS One. 2010 Jul 22;5(7):e11703. doi: 10.1371/journal.pone.0011703 (PMC2908537; doi:10.1371/journal.pone.0011703)

**Supplemental Figure S1**

**
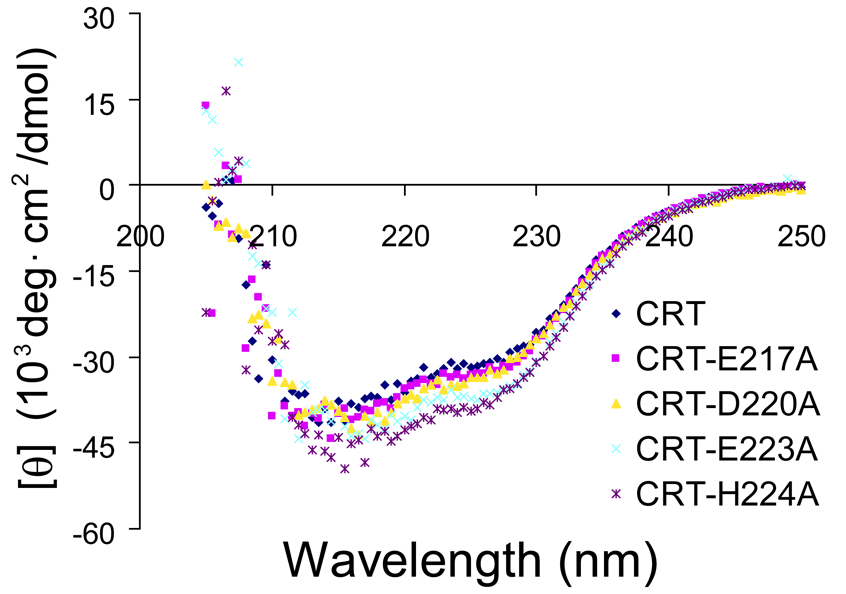
**

Supplement: Figure S1 — CD spectra of WT CRT and its mutants. Proteins, in a 5 mM HEPES (pH 7.4), 100 mM KF, 2 mM CaCl2 buffer were placed in 1 mm bandpass quarta cuvettes and analyzed by far-UV circular dichroism on an Aviv 215 spectropolarimeter (Aviv Associates, Lakewood, NJ). Corresponding baselines were obtained with buffer which were subtracted from the sample spectrum. No statistically significant conformational differences between WT CRT and its mutants were found. (0.26 MB DOC) [file pone.0011703.s001.doc]
