# Supplementary material for: Identification of the Rheumatoid Arthritis Shared Epitope Binding Site on Calreticulin
Source: PLoS One. 2010 Jul 22;5(7):e11703. doi: 10.1371/journal.pone.0011703 (PMC2908537; doi:10.1371/journal.pone.0011703)
Supplement: Table S1 — Primers for rabbit and mouse CRT site-directed mutants. (0.06 MB DOC) [file pone.0011703.s002.doc]

**Supplemental Table S1: Primers for rabbit and mouse CRT site-directed mutants**

|  | | |
| --- | --- | --- |
| rabbit CRT^E217A^ | Forward | 5’-CTCCAAGCCCGCAGACTGGGACAAGCCCGAG-3’ |
|  | Reverse | 5’-TGTCCCAGTCTGCGGGCTTGGAGTCCGTGG-3’ |
|  | | |
| rabbit CRT^D220A^ | Forward | 5’-GAGGACTGGGCAAAGCCCGAGCACATCCCCGAC-3’ |
|  | Reverse | 5’-GCTCGGGCTTTGCCCAGTCCTCGGGCTTG-3’ |
|  | | |
| rabbit CRT^E223A^ | Forward | 5’-GGACAAGCCCGCACACATCCCCGACCCGGAC-3’ |
|  | Reverse | 5’-CGGGGATGTGTGCGGGCTTGTCCCAGTCC-3’ |
|  | | |
| rabbit CRT^H224A^ | Forward | 5'-GGACAAGCCCGAGGCCATCCCCGACCCG-3' |
|  | Reverse | 5'-CGGGTCGGGGATGGCCTCGGGCTTGTCC-3' |
|  | | |
| rabbit CRT^Y254A^ | Forward | 5’-CAGAACCCCGAGGCAAAGGGTGAGTGGAAGCCGCGGCAG-3’ |
|  | Reverse | 5’-ACTCACCCTTTGCCTCGGGGTTCTGAATCACCGGCGGCTC-3’ |
|  | | |
| rabbit CRT^N279A^ | Forward | 5’-CGAAATCGACGCACCCGAGTACTCGCCCGACGCTAAC-3’ |
|  | Reverse | 5’-AGTACTCGGGTGCGTCGATTTCGGGGTGGATCCAGG-3’ |
|  | | |
| rabbit CRT^Y282A^ | Forward | 5’-CAACCCCGAGGCATCGCCCGACGCTAACATCTATGC-3’ |
|  | Reverse | 5’-CGTCGGGCGATGCCTCGGGGTTGTCGATTTCGGGGAC-3’ |
|  | | |
| mouse CRT^E217A^ | Forward | 5'-CAGATTCCAAGCCTGCGGACTGGGACAAGCC-3' |
|  | Reverse | 5'-GGCTTGTCCCAGTCCGCAGGCTTGGAATCTG-3’ |
|  | | |
| mouse CRT^D220A^ | Forward | 5'-CCTGAGGACTGGGCCAAGCCAGAGCAC-3' |
|  | Reverse | 5'-GTGCTCTGGCTTGGCCCAGTCCTCAGG-3' |
|  | | |
| mouse CRT^E223A^ | Forward | 5'-CTGGGACAAGCCAGCGCACATCCCTGACC-3' |
|  | Reverse | 5'-GGTCAGGGATGTGCGCTGGCTTGTCCCAG-3’ |
|  | | |
| mouse CRT^H224A^ | Forward | 5'-GGGACAAGCCAGAGGCCATCCCTGACCCTG-3' |
|  | Reverse | 5'-CAGGGTCAGGGATGGCCTCTGGCTTGTCCC-3’ |
|  | | |
